# Correspondence

**Published:** 1866-08

**Authors:** 


### Correspondence.

BUFFALO, August 6th, 1866.

*To the Editor of the Buffalo Medical and Surgical Journal:*

Dear Doctor:—Have the kindness to say, in your forthcoming number, that the case reported and published as proceedings of the Association on page 477, July number, was intended to be the report of a case of resuscitation, by the practice of Marshall Hall's method, prolonged for one hour and a half, during which time involuntary respiration was entirely suspended.

The case as reported is not much unlike the play of Hamlet, with the part of the principal actor omitted.

Yours, truly, C. C. F. GAY.

BOSTON, August 10, 1866.

*Editor Buffalo Medical and Surgical Journal:*

Dear Doctor:—You will perceive by the place from which this comes that I am back in old Boston, probably to remain here. But your journal has found its way to me, though I am away "down east." It came from Philadelphia.

I am glad that you "remember trying to abuse me out of the idea that I could cure epileptics." But your "*hope* that you did not fail in your efforts was a vain one." About the *cure*, however, I have a word to say. I have not used that term, nor do I pretend to do more than others ought to accomplish. From a somewhat extended experience, and in a practice that has brought me in contact with a large number of nervous diseases, I am authorized in saying that not more than one case in ten of epilepsy arises from *organic disease* of the brain, and from most of the nine-tenths from other causes, recoveries may be hoped for; indeed, if I did not think you would give me another *scolding*, I would say *expected*. Why should such not be the fact? One-half of them are caused by *self-abuse*. When this evil habit is abandoned, and proper means used to recuperate the system, (where nature has not been wholly destroyed,) means that are, or should be within the power of every physician, why should not such cases recover? If you can assign any reason, should like to know it. The same may be said in many cases arising from other causes.

Now, my dear sir, I have never been so foolish as to pretend to make a new brain; or, to restore an epileptic to health when the vital principle has become extinct; or, as some of you eminent *surgeons* do, take a man all to pieces, as you would an old watch, clean the parts well, and then put him together and make him as good as new.

If I remember correctly, among others, you had the case of Rev. C. A. Brucker, a Baptist clergyman from Charleston, S. C. That account he wrote and published himself. I never saw it till it was in print. It was a true account. I never said "I cured him." But he never had a fit after he came under my care. You may ascribe it to anything you please. After he had been free from fits two years, he said to me, "Dr. Cornell, I had but little expectation of recovery when I came to you. I had been under the care of some of the best physicians in Charleston, and I believe there are none better, and one of them the day I left, said to me, 'Mr. Brucker, you may go to Philadelphia, to Dr. Cornell, or any other doctor, but there is no power on earth that can cure you.'"

There it is, and it may pass for what it is worth. Had this been a solitary case, it might have been ascribed to a very fortunate *chance*, a mere coincident. But the case of Wm. F. Page of East Stoughton, Mass., was a similar one. He is still alive, in good health, and has not had a fit since 1848, though he had had them every three or four weeks for seventeen years previously. It is very easy to say, this was but another fortunate coincidence! Very well, all that is desirable is, to have these fortunate coincidences occur often enough; that is, as often as these cases come. But as you are a forbearing man, I will not inflict any more upon you about epilepsy at present.

I did not get a number of the journal in which my name was corrected. But as you suppose everybody knew my name was William M., being *so* notorious, perhaps it is just as well. I want to write a paper for your journal upon *Chronic Diseases*—their connexion with acute diseases; but I do not know when I shall find the time.

Will you send your journal to me at Boston, Mass.? Did you get the little book of mine called the "*Beacon;*" and, if so, did you notice it? I did not see the notice. You know when a man has been well flogged once, and deserves it, he is looking out for another. This book may open the way for it; so, if you have not yet received it, I will send you one.

Very truly yours, WM. M. CORNELL.

---

N. B., August 16, 1866.

*Editor Buffalo Medical and Surgical Journal:*

I have not troubled you in some time, and would not now, were it not about a matter of the utmost importance to the medical profession. It is a discovery of great moment to the Allopathic denomination, made on the Homœpathic theory of practice, and for which they deserve most of the praise. My experiments that led to the discovery, commenced shortly after reading a communication on the treatment of cholera in one of our daily journals, not long since, wherein it was gravely stated that camphor in saturated solution would give a person the cholera to his "heart's content," (not the place mentioned in connection with the Atlantic cable,) and consequently, and Homœphathically, *similia similibus*, etc., the saturated solution of camphor will cure the cholera. Now, Mr. Editor, taking that theory as a basis, I have been experimenting "some," and have had ample opportunities and subjects, there being *now* "so much cholera in *town*," but I will only give a brief history of one case at this time, for I am anxious to get the subject before the profession without loss of time, so that the people may have the full benefits of my new "treatment."

Case No. 1.—A German named John Crow, aged 45, a stout, healthy German laborer, had eaten young veal, boiled cabbage, and green corn, also some soured potatoes and cucumber salad; after which he had drank several quarts of beer during the evening. At one o'clock A. M. I found him in a cold, clammy perspiration, sunken eyes, surrounded with areola, skin shriveled, cramps in stomach and extremities, watery discharges, pulse 140, and feeble, calling for water, (which showed him to be partially delirious.) Now, Mr. Editor, there can be *no doubt* but that the combination of the animal and vegetable substances *produced* the disease. So on the theory that like cure, I immediately made the following prescription:

No. 1. ℞ Kalb Fleish.
Kraut.
Welch Korn.
Kroom Berren.
Kukumner Salat ein viertel pfund. M.

To be digested for half an hour in lager beer—ein gall. Take ad libitum.

But the symptoms were so very urgent, as a *placebo*, the following was administered:

No. 2. Pulv. Ipicac ʒ ss.

" Capsici gr. xx.

" Zingiber ʒ i.

" Ant. et Pat. Tart. gr. x.

Dissolved in a teacupful of warm water. Dose, tablespoonful every ten minutes.

During the time occupied in preparing ℞ No. 1, the patient vomited, filling a good sized tub with the contents of his stomach. The ordinary after treatment, such as opii, tanin, counter-irritation, heat and brandy was prescribed, and recovery followed. But in all similar cases I would recommend the profession to try ℞ No. 1.

A CONVERTED ALLOPATHIST.

